# Medical News

**Published:** 1852-04

**Authors:** 


### THE MEDICAL EXAMINER.

PHILADELPHIA, APRIL, 1852.

Several communications, and bibliographical notices, intended for this number, have been unavoidably postponed for want of room. We ask the indulgence of those who have sent their works to us for review.

### MEDICAL NEWS.

*Philadelphia County Medical Society.*—At the Annual meeting held on the 20th of Jan. last, the following were elected Officers of the Society for the current year:

*President*—Samuel Jackson, M. D.

*Vice-Presidents.*—John F. Lamb, M. D., and Isaac Parrish, M. D.

*Recording Secretary.*—D. Francis Condie, M. D.

*Corresponding Secretary.*—Henry S. Patterson, M. D.

*Treasurer.*—Wm. Byrd Page, M. D.

*Censors.*—E. F. Leake, M. D., N. L. Hatfield, M. D., Lewis Rodman, M. D., Wm. N. Johnson, M. D., Wm. Henry, M. D.

At an adjourned meeting of the Society held February 10th, 1852, the following were elected:

*Delegates to the American Medical Association.*—Samuel Jackson, (Professor), Benj. S. Janney, Lewis Rodman, Chas. D. Meigs, Robert A. Given, Chas H. Bibighaus, Thos. H. Yardley, Thos. F. Betton, Wm. H. Klapp, Isaac Parrish, D. Francis Condie, Wm. Darrach, Jno. D. Logan, and Jno. F. Lamb.

*To the Pennsylvania State Society.*—Isaac Hays, Geo. B. Wood, Wm. Pepper, Geo. Fox, Samuel Jackson, Geo. W. Norris, Paul B. Goddard, John F. Lamb, A. Naudain, Samuel Jackson (Professor), G. Emerson, Henry Bond, Alfred Stille, Thomas D. Mütter, Jos. Pancoast, Wm. Mayburry, D. Francis Condie, Henry S. Patterson, Francis West, Isaac Remington, I. M. Pugh, Thos. F. Betton, Wm. R. Grant, Joseph Warrington, C. H. Bibighaus, Wm. Henry, Edward Janvier, and G. W. Patterson.

It was *Resolved,* that should any of the Delegates thus elected be unable to attend the approaching Session of the American Medical Association or State Society, or if he has been elected a Delegate from any other body to the former, he be requested and empowered to appoint some other member of this County Society as shall be able to attend as his substitute, and that such appointment, set forth in writing, and signed by the Delegate by whom it has been made, shall serve as the credential of said substitute.

Delaware County Medical Society.—Officers elected for 1852.

*President.*—Dr. Jesse Young, Chester.

*Vice-President.*—Dr. S. A. Barton, Aston.

*Secretary.*—Dr. Robert K. Smith, Darby.

*Corresponding Secretary.*—Dr. George Martin, Concord.

*Treasurer.*—Dr. M. Emanuel, Marcus Hook.

*Delegate to the American Medical Association.*—Dr. Robt. K. Smith, Darby.

*Delegates to the State Society.*—Drs. Smith, Emanuel, Martin, and Barton.

Delegates to the American Medical Association, appointed by the College of Physicians of Philadelphia.—John Rodman Paul, G. Emerson, Francis West, Alfred Stillé, John Neill, W. S. W. Ruschenberger, S. L. Hollingsworth, Geo. B. Wood, Caspar Wister, Isaac Hays, Rene LaRoche, John D. Griscom, John B. Tuft, Francis G. Smith, Anthony E. Stocker.

Physicians' Prescriptions.—The report of the joint committee of the Philadelphia County Medical Society and Philadelphia College of Pharmacy, on this subject, published in our number for January last, was unanimously adopted by the County Society at its Annual meeting held January 20th, 1852, with this proviso:

"That nothing therein contained shall be construed into any sanction or countenance, direct or indirect, on the part of this Society, of the manufacture, sale, or use, by any one, or under any pretext, of quack or secret medicines."

Appointment in the University of New York.—We have great pleasure in announcing that the chair of Anatomy in the University of New York, vacated by the death of Dr. G. S. Pattison, has been filled by the appointment of Dr. Wm. H. Van Buren; a gentleman admirably calculated to occupy, with credit to himself and profit to the school, so distinguished a post.

We make this announcement with the more pleasure, as it enables us to correct an unintentional error in our last number, in regard to the *rumor* that application had been made to a gentleman in this city, prior to Dr. Van Buren. We have the best reason for believing this to be a mistake. Dr. Van Buren was the first choice after Dr. Pattison's death. Our information was derived, in the first instance, from a New York daily paper.

Itch Insect.—Most of our readers must have seen the *Acarus scabiei* under the microscope, or are at least acquainted with the figures of the creature which have been published. It appears that the male insect has, until lately, eluded observation, but has been at last dragged into light by M. Lanquetin, of Paris. The amiable parasite is described in the "Union Médicale," and is said to be only half the size of his better half.

Spread of Disease and fever in Ireland.—In our last we felt it our duty to allude to the alarming increase of contagious and infectious diseases amongst the poor in Ireland. We regret to find that our worst fears are likely to be realized, and that there is every prospect of that ill-fated land being further made desolate by fever, engendered by famine and neglect. According to the last report of the physicians of the South Dublin Union, the state of that workhouse is frightful to contemplate. Those gentlemen say that, in addition to the usual disease contingent on the season, the house has been visited by severe epidemic suffocative catarrh, also by a new form of febrile epidemic, in addition to a large amount of measles. They also reported forty-six cases of a malignant character, and that in the sick and infirm wards, the patients amounted to the formidable number of 1371. In consequence of this extraordinary increase of disease, the physicians have demanded additional help.—*London Lancet, Feb.* 14*th*, 1852.

American Medical Association.—The fifth annual meeting will be held at Richmond, Va., on Tuesday, May 4th, 1852.

All secretaries of societies, and of other bodies entitled to representation in this association, are requested to forward to the undersigned correct lists of their respective delegations as soon as they may be appointed.

The following is an extract from Art. II. of the constitution.

" Each local society shall have the privilege of sending to the association one delegate for every ten of its regular resident members, and one for every additional fraction of more than half this number. The faculty of every regularly constituted medical college or chartered school of medicine shall have the privilege of sending two delegates. The professional staff of every chartered or municipal hospital containing a hundred inmates or more, shall have the privilege of sending two delegates; and every other permanently organized medical institution of good standing shall have the privilege of sending one delegate."

The medical press of the United States is respectfully requested to copy. P. Clairborne Gooch, *One of the Secretaries*,
Bank-street, Richmond, Va.

Discontinuance of the British American Medical and Physical Journal.—We learn, with much regret, that this excellent Journal published at Montreal, Canada, under the able editorial conduct of A. Hall, M. D., has been discontinued.

Etherization in Parturition Nineteen Years ago.—Dr. Walter Channing reports in the Boston Medical and Surgical Journal, for March 10th, 1852, the following interesting details on this point. Dr. Channing was recently attending a case, in which he administered ether with "excellent effects." In his visit following the delivery, he asked his patient "how many times she had used ether. She named them, and added that there was one other time in which she used it with great advantage. I asked when. Nineteen years ago, she said, she gave birth to her eldest son. Her labor lasted more than a fortnight. In the absence of her physician, her husband tried to find something which had given some relief in her former and first labor. He failed; but being engaged in preparing a chemical lecture, and making experiments with sulphuric ether, he thought he would try that. It was wiped freely over her face, and forehead, and *breathed*. To his surprise all her distress passed away—the spasmodic twitchings disappeared—violent voluntary effort, and which constituted so much of her misery then, and has in all subsequent labors, ceased to annoy her. Her physician arrived, and was so much pleased with the effects of the ether that it was employed during the rest of the labor. Her labor was now easy, was soon completed, and a stout living boy born. Such was her account of her first use of sulphuric ether to diminish or to abolish pain."

Medical Circulars.—Such is the competition in medical instruction, in this country, that even the schools of medicine of some of the States appear to be earnestly drumming up the people for customers. Circulars are actually flying through the post offices, announcing the facilities, economy, &c., of certain institutions for next November. This is taking time by the forelock; but it is positively injuring the medical character of the United States, to make such a show of ambition to gather up students.—*Boston Med. and Sur. Journ. 10th March.*

Remarkable Case of Inanition.—The following extraordinary occurrence is detailed in a late number of the Kingston (Ky.) Republican:

"On the 29th ult., a negro woman belonging to Mr. J. Harpending, of this County, got lost in the woods. Mr. H., thinking she had been stolen, offered a reward for her. He heard nothing of her until the 11th inst., when some boys who were hunting, found her, apparently dead. They returned home, and informed some gentlemen of the fact. Messrs. B. W. Harpending, E. George and one or two others, went in search of and found her, almost covered with snow; and supposing, as a matter of course, that she was dead, one of the party started to get a slide, while the others struck up a fire and awaited his return. One of them, wishing to see if decomposition had taken place, touched her with his cane, when, to his astonishment, she slightly moved her head. After applying the usual remedies she recovered sufficiently to converse with them. She stated that she had not eaten or drank anything but snow since she left home, and had been out in the weather all the time—fourteen days. She is in a fair way to recover. When she left home she was very fleshy, but when found was perhaps the most emaciated creature ever seen alive. These facts can be substantiated by the testimony of some of the most respectable men of our county."

University of Louisville Law Suit.—We learn with pleasure, that a suit pending between the City of Louisville and the Board of Trustees of the University, has been decided in favor of the latter. A provision of the new City charter had given the election of the Board of Trustees "to the people," but the Court has decided this to be unconstitutional, and "the administration of the school remains in the hands where it was."

Resignation of Dr. John Bell.—Our esteemed townsman has resigned his Chair of Theory and Practice in the Medical College of Ohio, and returns to Philadelphia at the close of the present term.

Obituary.—We announce with deep regret the sudden death of Dr. Wm. R. Grant, *Professor of Anatomy in Pennsylvania Medical College.* Dr. Grant, by his amiable deportment, high professional and moral worth, had secured the esteem of all who knew him. His loss will be deeply felt by a large circle of friends, and by the School of which he was a valued member.

### NEW LIGHTS AND NEW LIVERS.

"When music, heavenly maid, was young,"
Rudely in ancient Greece she sung,
Now modern Grease usurps the song,
And smooths the rugged verse along.

In former times before the day
Of wisdom dawned upon our way,
And gave the medical profession
Her highest gifts in full possession,
Granting these choice Disciples' skill,
To bind on earth, by draught and pill,
And not to loose, as might befall,
This erewhile chosen Israel,
Before in chorus had been sung
"Jam Satis," ancient Fogydom,

Consumption, as tradition runs,
Made man expectorate his lungs;
Untaught there lay, by grace of God,
Salvation for him in a Cod;
Unmeet, the mystery to explain,
O'er which Theologians rack'd the brain,
How stretched upon Samaria's field,
His very soul in act to yield,
The dying traveller was restored,
When o'er his wounds the oil was poured.

Untaught that here, in meaning full,
Was hidden prophetic parable,
Which Providence would us inspire
To read by inspiration higher
Than e'er Joe Miller read Isaiah;
That figuratively fallen in strife,
'Twas Phthisis threatened here a life,
And fled when oil the wounds had dressed,
"Genuine cod liver, cold expressed."

And little thought those rugged hands,
That, round Newfoundland's stormy sands,
From Ocean's depths drew forth their prey,
All struggling to the light of day,
That Irish girls might harmless dine,
Spiritual and temporal combine,

To cheat Satanus of his toll,
By Friday's strengthening of the soul,
That they were adding to heaven's Will
An unexpounded Cod-icil.

Nor dreamed each fish at middle bore,
(When science taught us to explore,)
Both lives and liver for their turn,
Like archers true at Bannockburn,
That there displayed in full essence,
Was fount of rejuvenescence,
Which dull De Soto would have laid
In Florida's dark everglade;
When, heaven inspired, he might have drank,
Rich purling from Newfoundland's bank.

Unchanged as food, now altered its effect,
Now used by election, once by the elect;
Then, "Aid us Maria, in all ill,"
Now Gadus Morrhua be our fill,
And erring man, with all his hopes at stake,
Is once more aided by an act of faith,
And Vomicas which, in their day,
Resounded with the "pôt fêlé,"
All teach auscultors to admire,
The "cracked pot sound" one story higher.

E're struggling infants from the womb
Their circulation can resume,
(Provided they've not come too soon,)
The attending surgeon seeks the loin,
And carefully explores the groin,
And if one gland he can discover
A hair's breadth fuller than another,
No modern surgeon here would miss,
Tuberculous Diathesis,
And straightway at his solemn nod,
The infant's suckled by a Cod.

Hail modern Grease, be thine a fame,
More than thy prototype could claim,
While modern doctors they confess,
Extremâ cerâ Cod-icis. YOUNG PHYSIC.

